# Understanding the role of the state in dietary public health policymaking: a critical scoping review

**DOI:** 10.1093/heapro/daad100

**Published:** 2023-09-04

**Authors:** Nancy Karreman, Yuru Huang, Natalie Egan, Lauren Carters-White, Benjamin Hawkins, Jean Adams, Martin White

**Affiliations:** MRC Epidemiology Unit, University of Cambridge School of Clinical Medicine, Cambridge, UK; MRC Epidemiology Unit, University of Cambridge School of Clinical Medicine, Cambridge, UK; MRC Epidemiology Unit, University of Cambridge School of Clinical Medicine, Cambridge, UK; SPECTRUM Consortium, Usher Institute of Population Health Sciences and Informatics, Centre for Population Health Sciences, University of Edinburgh, Edinburgh, UK; MRC Epidemiology Unit, University of Cambridge School of Clinical Medicine, Cambridge, UK; MRC Epidemiology Unit, University of Cambridge School of Clinical Medicine, Cambridge, UK; MRC Epidemiology Unit, University of Cambridge School of Clinical Medicine, Cambridge, UK

**Keywords:** diet, food, health policy, qualitative methods, systematic review

## Abstract

Despite evidence that dietary population health interventions are effective and widely accepted, they remain the topic of intense debate centring on the appropriate role of the state. This review sought to identify how the role of the state in intervening in individuals’ food practices is conceptualized across a wide range of literatures. We searched 10 databases and 4 journals for texts that debated dietary population health interventions designed to affect individuals’ health-affecting food practices. Two co-authors independently screened these texts for eligibility relative to inclusion and exclusion criteria. Thirty-five texts formed our final corpus. Through critical reflexive thematic analysis (TA), we generated 6 themes and 2 subthemes concerning choice, responsibility for health, balancing benefits and burdens of intervention, the use of evidence, fairness, and the legitimacy of the state’s actions. Our analysis found that narratives that aim to prevent effective regulation are entrenched in academic literatures. Discourses that emphasized liberty and personal responsibility framed poor health as the result of ‘lifestyle choices’. Utilitarian, cost-benefit rationales pervaded arguments about how to best balance the benefits and burdens of state intervention. Claims about fairness and freedom were used to evoke powerful common meanings, and evidence was used politically to bolster interests, particularly those of the food industry. This review identifies and critically analyses key arguments for and against population dietary public health policies. Our findings should motivate public health researchers and practitioners to avoid unreflexively embracing framings that draw on the languages and logics of free market economics.

**Contribution to Health Promotion**

- Health promotion activities intervene in practices generally considered to be personal or private, including diet.
- We searched academic papers for arguments about whether and how the state should intervene in diet.
- We identified arguments across six key areas: individual choice, responsibility for health, balancing costs and benefits, evidence, fairness, and whether the state’s actions are legitimate.
- Many arguments we identified used economic language and methods that can be used to delay or prevent regulation of the food industry.
- We caution the public health community against unconsciously drawing on these arguments and implicitly supporting the diversionary messages of the food industry.

## INTRODUCTION

Less healthy foods including those that are energy-dense and high in saturated fat, sugar, and salt; are often cheap; heavily promoted; and readily accessible. In the UK alone, adults consumed 66% more sugar and 40% more salt than recommended in 2019 (NatCen Social Research and NIHR Biomedical Research Centre, 2020). About 15% of all life years lost in the UK in 2018 were linked to poor diet (Steel *et al.*, 2018) and progress in addressing this has been slow (OHID, 2022). Rose (Rose, 2008) theorized that population approaches are more effective than those that target high-risk groups. Politically palatable, advice-based interventions that require individual recipients to make use of their personal resources, or agency, to benefit may entrench social inequalities and fail to acknowledge how the unequal distribution of economic power across society modulates access to resources (McCartney *et al*., 2021). Alternatively, structural interventions to change food environments (Dimbleby, 2021), such as the UK Soft Drinks Industry Levy (SDIL), may be more effective and equitable (Adams *et al*., 2016).

Despite this, structural interventions are often accused by libertarians and by those aligned with industry interests of ‘nanny statism’—that is, that these measures inappropriately limit individual liberty and remove personal responsibilities in ways that are morally corrosive. In the case of food this position has been challenged as relying on an implausible account of autonomy (Wilson and Dawson, 2010) that neglects the influence of genetic (Loos and Yeo, 2022) and socio-environmental factors (Caspi *et al*., 2012). The growing field of commercial determinants of health research has begun to reckon with the complex relationships between commercial actors and health outcomes (Gilmore *et al*., 2023). Like the tobacco industry, food manufacturers use a ‘playbook’ of strategies to challenge evidence, lobby for less restrictive regulation, and deny the harmful nature of some of its products (Brownell and Warner, 2009). Any effort to intervene in dietary public health (DPH) must take this context into consideration.

There are myriad frameworks for understanding the appropriate role of the state emanating from public health, medicine, philosophy, policy studies, law, economics, and sociology. However, this body of work has not been previously reviewed and synthesized across disciplines [for an ethics review, see (Hurlimann *et al*., 2017)]. This article presents the findings of a critical scoping review that critically assesses key arguments for and against population DPH policies across disciplines as they relate to the role of the state. Our aim was to describe, in a systematic and replicable way, the scope of research activity on a previously underexplored topic and to summarize and disseminate existing research findings (Arksey and O’Malley, 2005; Peters *et al*., 2015). We applied a critical lens to our analysis because it provided an opportunity to take stock of, and critically evaluate, important academic discourses stemming from disparate literatures at the intersection of public health, policy studies, and social sciences (Grant and Booth, 2009).

## METHODS

This paper reports part of a review that addressed the question: How is the role of the state in developing and implementing population public health policies designed to influence individuals’ health-affecting practices explained in relevant academic literature? Referring to health-affecting practices is an intentional move away from the predominant framing of ‘lifestyle choices’ and towards a socioecological understanding of health-related personal practice (Olstad and Kirkpatrick, 2021). A protocol outlining the original rationale, search strategy, and inclusion criteria was uploaded to Open Science Framework (OSF) in January 2021 (Karreman *et al*., 2021). We report here a review of a subset of included texts specifically focussed on DPH according to Preferred Reporting Items for Systematic Reviews and Meta-Analyses (PRISMA) guidelines (Tricco *et al*., 2018), detailed in Supplementary File 1.

### Search strategy

We used a standard search strategy in line with guidance published by the Joanna Briggs Institute (JBI) and PRISMA Extension for Scoping Reviews (PRISMA-ScR) (Rethlefsen *et al*., 2021). The search strategy aimed to locate both published and unpublished (grey) literature.

We conducted systematic searches of five databases in January 2021 with no time limits: Scopus, Web of Science, MedLine, PhilPapers, and PsychINFO. These were supplemented with searches of grey literature repositories ProQuest, OSF Preprints, SSRN, PhilSci Archive, and WorldCat; Google and Google Scholar searches; and hand-searching of four journals in February 2021. The complete search strategy can be found in Supplementary File 2.

### Selection criteria

For a text to be included, the role of the state in making public health policy must have been referred to in the title or abstract (eligibility screening) and expanded upon in the full text (inclusion screening) and primarily concern attempts to influence individual health-affecting practices (e.g. mandatory vaccination, seatbelt wearing). The state is understood as the government and public sector system, including institutions and actors that make decisions that affect the population. Sources that focussed on evaluating the efficacy of single interventions, or on a high-risk sub-population (e.g. prisoners) were excluded. Articles not in English and mass media items were also excluded.

### Screening

NK uploaded de-duplicated texts to Covidence, where they were screened independently by NK and either YH, NE, or LCW in two rounds by applying the selection criteria to each text’s title and abstract (or equivalent) and full text. In the case of books or long material, we used an abstract proxy to aid screening: if after an examination of the introduction, table of contents, chapter summaries, and index, the source did not appear likely to meet inclusion criteria, it was excluded. Any disagreements that arose between the reviewers at each stage were resolved through discussion, or with reference to an additional reviewer (MW). Our screening process is documented in Supplementary File 3.

### Data extraction

We used a standardized extraction form to collect information about each included text including title, authors, date, publication disciplinary affiliation, main topic, and a summary of relevant content. The judgement of disciplinary affiliation was made on the basis of the publication and authors’ research areas. The extraction form was piloted by NK and YH and implemented by NK. We also judged whether the text provided a *thick* or *thin* contribution to addressing our question (Ponterotto, 2015): whether there was sufficient propositional content on the key focus of the review to warrant inclusion in the first round of data coding, reaching resolution through deliberation when any disagreements arose. We narrowed our analytic focus to included texts addressing DPH at this point.

### Thematic analysis

NK led the thematic analysis (TA) as described by Braun and Clarke (Braun and Clarke, 2022). This involved familiarization with the dataset; iterative inductive data coding with the assistance of NVivo software; generating initial themes; developing and reviewing themes; refining, defining, and naming themes; and writing up. NK prepared preliminary themes from patterns across the dataset and refined them through continuous discussion with the rest of the research team. This formed a core part of our reflexivity practice, further detailed in Supplementary File 4.

We focussed initially on coding ‘thick’ texts, then sampled randomly from ‘thin’ texts until sufficient ‘information power’ had been reached (Malterud *et al*., 2016) to address our compass question. We used information power, rather than saturation, as a target for breadth and depth of coverage in our corpus because it steers away from a (post-)positivist conception of saturation as ‘an absolute status that can be conclusively achieved’ and towards a recognition of whether we had reached a significant depth of analysis (Varpio *et al*., 2017, p. 45). We did not ultimately carry out reference searching or consult extensively with experts on our final included corpus because we felt that we had already reached sufficient information power for thematic depth.

## RESULTS

After two rounds of screening, we generated a corpus of 255 texts meeting our inclusion criteria. We then narrowed our scope to focus on texts that included explicit references to dietary practices, resulting in a total of 53 texts (30 ‘thick’ and 23 ‘thin’). Our final corpus for this review (Supplementary File 5) consisted of all 30 ‘thick’ texts and 5 ‘thin’ texts that were added through random sampling. Texts ranged from discussions about the legitimacy of public health generally to specific arguments for and against, for example, sugar-sweetened beverage (SSB) taxes. Our analysis generated 6 themes and 2 subthemes (Supplementary File 6) based on close textual analysis. Exemplar quotes and associated references are included in Supplementary File 7 and indicated via numerical citations in the main text.

### The state should not intervene in choice

This theme reflects claims that the universal value of liberty in Western societies must be preserved, even if it is realized and protected differently in different contexts. Under the liberal paradigm, state paternalism is unacceptable because it substitutes the judgement of the state for that of the individual and violates their right to self-governance; curbing individual freedom is only permissible to prevent harm to others (Mill, 1859). Consequently, the state can only legitimately moderate an individual’s autonomy under highly circumscribed conditions that require rigorous justification. This paradigm is embedded in the influential (Nuffield, 2007) ladder, which ranks public health interventions by their degree of infringement on individual liberty.

Interventions that provide information to consumers, like education or food labelling, are justified in part by their low impact on liberty whereas taxes and advertising restrictions are classed as more intrusive and therefore less acceptable (see Supplementary File 7 [1, 2]). The claim that more information will result in ‘better’ choices relies on an idealized vision of the market, and the individual as a rational actor [3], that fails to account for the power of commercial actors and the influence already exerted on people by their environment. Coggon and Adams (Coggon and Adams, 2021) argue that interventions that rely on this model of individual deliberation are ineffective and inequitable. Instead, we should prioritize lower agency interventions that target changing the environment in which individuals live [4]. Dietary practices are also described as not fully autonomous because they are habitual [5], pose risk to the agent themselves [6], and are not truly free [7]. Intervention in choices that are not truly autonomous is perceived to be more ethically justifiable and is much less controversial (Barnhill *et al*., 2014). Appeals to freedom are also used to justify public health measures by arguing that interventions broaden freedom by resolving information asymmetries (Resnik, 2014) or increasing options (Falbe, 2020) and the capabilities of individuals to pursue them (Shrimpton, 2003; Herington *et al*., 2014; Buchanan, 2015; Veliz *et al*., 2019).

However, Resnik (Resnik, 2010) argues that, by adopting policies that ostensibly limit individual liberty, we risk falling down a ‘slippery slope’ that would enable intrusive government control of personal practices [8]. Contrastingly, Veliz *et al*. (Veliz *et al.*, 2019) point out that the state is held to a higher standard of non-interference than industry, though both seek to influence individuals [9]. Falbe (Falbe, 2020) argues that if it is ethical to pursue certain corporate strategies to sell more products, then it should be ethical for the state to employ alternative strategies to sell fewer [10]. Kaldor (Kaldor, 2018) also notes that arguments that government regulation infringe liberty are not applied to industry actors, despite the latter also possessing and maintaining considerable power over individual choice and behaviours.

### Responsibility for health

The justification for state intervention in the interest of DPH depends on the attribution of responsibility for public health to individual ‘choices’ or wider contextual and socioeconomic factors. Personal responsibility narratives underpin more individualized, ‘downstream’ approaches whereas structural issues narratives suggest more environmental, ‘upstream’ approaches (Adams *et al*., 2016). Herington *et al*. (Herington *et al.*, 2014) hold that individuals cannot be conceived as having ‘chosen’ to become obese due to the complex interaction between social conditions, like socioeconomic status and educational attainment; genetics; and autonomy. State intervention is thus legitimated on the basis of conflict between an individual’s presumed interest in living a healthy life and social conditions that increase the risk of obesity [11].

An account of personal responsibility might proceed as follows: since the choices that cause poor outcomes are chosen freely and rationally by individuals, they are therefore responsible for these outcomes and they may not be an issue that other actors including the state or food industry should remedy [12]. Additionally, this might harm individuals’ health literacy and agency [13]. In either case, there is no obligation for state intervention, or it may even be wrong to intervene, because poor health outcomes are the result of rational choices.

Whether and what kind of government intervention is required depends on whether dietary practices and their associated health outcomes are considered ‘public’ problems. If dietary practices are solely private issues and matters of personal responsibility, then state intervention requires strong justification, whereas for public problems, less strenuous justification is necessary. Some authors claim a problem is made public through its prevalence (Herington *et al*., 2014; Brooks, 2015; Mayes, 2015; Grummon *et al*., 2020); is caused by structural factors outside of individuals’ control (Mello, 2012; Simões, 2013; Kass *et al*., 2014); or affects public goods, like healthcare, that are stewarded by the state (Creighton, 2009; Falbe, 2020; Anaf *et al*., 2021).

This theme permeates two, at times seemingly mutually exclusive mechanisms of action that arise from consideration of who is responsible for health outcomes: *the state should protect the public* and *the state should partner with industry*. The former considers the rationales that motivate an interventionist, protective state—to protect the public interest, rights, and groups—while the latter concerns what the relationship of the state with industry should be.

#### The state should protect the public

In this role, the state is described as having a duty to protect and promote health, especially of vulnerable groups, like children. The state must also respect and promote personal rights, which may at times conflict with population health. This subtheme also encompasses discussion of how health should be prioritized relative to other values like liberty and privacy.

The status of health as a social value is contentious; Resnik (Resnik, 2014) argues that individuals may not all be willing to make the same trade-offs for health, and that public health should be balanced against other considerations [14]. The public interest can be conceptualized in two opposing ways: as promoting the welfare of citizens or protecting the market economy and freedoms. Brooks (Brooks, 2015) argues that the latter model unhelpfully enables the food and drinks industry to deflect responsibility onto individuals and countervenes the duty of the state to promote its citizens’ health [15]. The state is in a better position to make health-promoting decisions than individuals, in terms of both judgement and resources, and so should intervene to promote our long-term interest in health.

Rights-based discourses also provide justifications and curbs on state action. Calls for the state to respect human rights characterize proper and improper modes of governing (Shrimpton, 2003; ten Have *et al*., 2011, 2013; Coggon and Adams, 2021; Ó Cathaoir, 2017). Tirosh (Tirosh, 2014) argues that a ‘fat tax’ levied on people living with obesity when purchasing calorie-dense foods would be unjustifiable on both moral and legal grounds because it infringes personal rights [16]. Framing proposals as promoting individual rights can also enable intervention, as in the case of the framing of childhood obesity. Authors argue that children are especially vulnerable to advertising because they are not yet capable of critical assessment [17] and are not yet fully autonomous, so their rights to autonomy may be restricted [18]. The state also has an interest in obesity prevention and treatment as part of a shared responsibility with parents and society to protect future citizens [19]. Narratives about childhood make policy problems more amenable to certain kinds of interventions, even those that are targeted not solely at children like the Soft Drinks Industry Levy (SDIL): the UK government’s 2016 obesity strategy framed measures including the SDIL as addressing the problem of ‘England’s rate of childhood obesity’ (Department of Health and Social Care *et al*., 2016).

Like rights discourses, appeals to morality and international norms justify intervention by regarding the state as having obligations to the public. Solidarity, described as the moral duty to prevent suffering of others in a community by Mello (Mello, 2008), obliges the state to alleviate the suffering of community members living with obesity [20]. Governments are also obliged by frameworks like the UN Convention on the Rights of the Child to provide certain protections to vulnerable groups [21]. Protecting rights therefore provides a basis for intervening in a market that otherwise should be minimally regulated.

#### The state should partner with industry

This subtheme centres on the relationship between the food industry, the market economy, and the state. The default position in many market economies is that the state should interfere as little as possible in the functioning of the market. Businesses are considered to have legitimate economic interests that make curbing their sometimes health-harming behaviours difficult. To enable intervention, the language of ‘market failure’ (Khemani and Shapiro, 1993, p. 55) has been steadily imported into the public health lexicon to account for issues such as childhood obesity. Proposed mechanisms for change substitute legislation and state authority with voluntary guidelines and corporate dominance (Kaldor, 2018). Public health attempts to legitimise interventions by tapping into the language of economics, and thereby accepting its underlying assumptions.

The logic of partnership between the industry, the state and the public positions industry actors as being part of the solution to public health problems. Falbe (Falbe, 2020) states that SSB taxation incentivises reformulation and responsibilities the industry to be ‘part of the solution’ (p. 5). Discourses of industry responsibility underpin voluntary and partnered approaches at the expense of more effective forms of external regulation. Those in favour of voluntary mechanisms argue that partnership approaches are effective, obviating the need for regulation [22]. Others, however, reject voluntary initiatives as ineffective or incompatible with public health goals (Moodie *et al*., 2013; Capewell and Capewell, 2018; Knai *et al*., 2018), promoting a more interventionist role for the state [23].

Reformulation is an interesting exception to the assertion that voluntary measures by industry are ineffective and serve industry interests. That the industry may choose or be pressured to reformulate its products rather than trigger compulsory measures is seen to be a key leverage point for partnership to promote public health (Yang and Nichols, 2011; Pratt, 2015; Kaldor, 2018; Veliz *et al*., 2019; Falbe, 2020; Grummon *et al*., 2020). This middle ground may be both more politically palatable and perhaps normatively so, as a behavioural nudge is more defensible from a libertarian perspective than an obligation.

### The state must balance benefits and burdens

This theme explores the idea that the benefits of public health interventions must outweigh any associated burdens. Though this may seem obvious, the definition of how benefits, burdens, and the balance between them reflects broader assumptions about the role of the state as a utilitarian decision-maker that must decide whether a policy is ‘worth it’ or not. This was sometimes expressed as *proportionality*: that the size or scope of intervention, and evidence to support it, should be tailored to the severity of the problem (Resnik, 2010; Kass *et al*., 2014; Pope, 2014; Brooks, 2015; Morain, 2015; Priest, 2015; Kaldor, 2018; Coggon and Adams, 2021). This principle is also formally incorporated into many core public health texts (Nuffield Council on Bioethics, 2007). The role of the state is also to reduce costs, aligning this theme firmly with economic logics and methods, including cost-benefit analysis.

The problem of widespread obesity in the population is generally framed as either a health issue; or as an economic issue associated with costs, impacts on economic productivity, and negative externalities (Creighton, 2009; Tirosh, 2014). Healthcare costs are used to illustrate the societal burden of obesity and to justify state intervention to reduce (financial) harm to the collective (Yang and Nichols, 2011; Kass *et al*., 2014). The state has legitimate interest in addressing problems that create collectivized costs: that the productivity of the workforce is decreased by ill health (Mello, 2012; Herington *et al*., 2014; Falbe, 2020); that healthcare costs are too high [24]; or that taxes generate revenue for government (Sassi *et al*., 2014).

Reducing costs appears to attract more focus than proposed benefits; this may reflect an understanding that public health benefits must be evidenced to overcome the default setting of non-action (or partnership) whereas potential burdens can be demonstrated by ethical and other reasoning. Grummon *et al*. (Grummon *et al.*, 2020) frame their final verdict on the ethical defensibility of food warning labels as dependent on costs and benefits [25]. Cost-benefit analysis has become an established practise for governments and academic analyses, fuelling claims that public health regulators should use cost-benefit analyses to rationalize their actions [26].

A subsidiary requirement of proportionality outlined by Kass *et al*. (Kass *et al*., 2014) is that higher quality evidence is required to justify increased burden to individuals, industry, or the public purse [27]. Though this is a relatively straightforward logic, there is no definitive threshold for when evidence has reached a sufficient level to justify a certain set of costs. For example, Resnik (Resnik, 2010) objects to *trans*-fat bans on the basis that their benefits are under-evidenced relative to the limitations they place on the liberty of individuals and businesses. Before resorting to bans, he argues, it must be established that they are more effective than education-based interventions. This creates a chicken-and-egg paradox when it comes to population-level public health interventions: there is not enough evidence to justify intervention, yet to generate evidence requires policy experimentation (Ogilvie *et al*., 2020). Public health researchers themselves acknowledge this difficulty and acknowledge the difficulty of proving cost-effectiveness [28], though surprisingly do not make use of the precautionary principle to argue for a bias toward prevention in the face of scientific uncertainty. This objection provides a powerful stall to *any* kind of public health intervention.

### The state must provide evidence of efficacy

This theme concerns the use of evidence to establish both public health problems and their proposed solutions. The goal of evidence-based policy is to overcome the barrier that politics presents to the effective use of research evidence (Hawkins and Parkhurst, 2016). Our included texts explicitly called for policy to be evidence-based, a call to a neutral consideration of ‘the facts’ and the primacy of science over politics. Kass *et al*. (Kass *et al*., 2014) suggest that a strong commitment by government to evidence-based policy is an imperative of social justice [29]. However, evidence and its presentation are never apolitical. The framing of evidence, even the same empirical statistics, is a deeply political practice that serves to advance certain interests and goals.

Disputing evidence as biased or unclear, weighing mixed evidence, and considering the effects of potential unintended consequences are all forms of political debate that are reflected in our included texts. Presenting evidence of the scope of an issue serves to try and attract attention to it and move it forward on the policy agenda whereas framing potential solutions as ineffective on the basis that evidence is unclear, contradictory, biased, or insufficient (Philipson and Posner, 2008; Simões, 2013; Wilkinson, 2019); that the problem is already resolving itself (Resnik, 2014; Tirosh, 2014); that there is no causal evidence linking the problem to the proposed solution (Pope, 2014; Resnik, 2015); or that the problem is too complex to be addressed by policy (Simões, 2013; Mayes, 2015) advances interests that seek to delay or make infeasible particular interventions. Claims about evidence are framed differently depending on the goal being advanced: as an ineffective silver bullet or as part of a broader policy agenda [30].

Evidence takes a variety of forms, including health and economic statistics; expert testimony; controlled trails; and evaluations of similar, previously implemented interventions. Expert endorsement of an intervention was treated as a proxy for the strength of evidence supporting the implementation of policies by some texts (Brooks, 2015; Anaf *et al*., 2021). Evidence of public support, such as that studies generally find high public support for food warnings (Grummon *et al*., 2020), was also presented as evidence supporting or opposing a policy in addition to its political legitimacy. The latter is explored further in the theme *the state’s actions must be legitimate*.

Arguments against intervention might mix evidence claims about the ineffectiveness of a previous policy alongside assertions that the proposed intervention would reduce personal liberty. If anything used to ground a claim can be characterized as ‘evidence’, then potentially limitless forms of evidence exist to suit the point one is trying to make. Arguments that more evidence is needed, such as those that Resnik (Resnik, 2010) makes about bans on trans-fats, remain vague about the threshold of evidence required, effectively discouraging intervention indefinitely.

### The state must act fairly

The ambiguous use of ‘fairly’ in the name of this theme reflects its multifaceted deployment in included texts. Fairness may refer to equality, as in equal treatment of individual persons, both natural and corporate. It may also gesture towards equity, usually taken to mean the absence of systemic disparities between groups. Social justice, or the promotion of equity, is traditionally framed as one of the two foundational pillars of public health (Beauchamp, 1976). Remedying health inequities provides a raison d’etre for public health and is a useful frame to motivate and justify public health interventions. Fairness is also evoked by arguments in opposition to public health interventions through labelling them as ‘unfair’ or ‘discriminatory’ in their effects. Some authors highlight the perceived inequ(al)ity that an intervention would create as a reason to do nothing, or to suggest an alternative solution [31].

Education and information campaigns are offered as legitimate alternatives, as according to this narrative, they do not impose more on disadvantaged groups. However, as explored earlier, information-based interventions alone may present more demands on an individual’s agency and inadvertently reproduce inequitable outcomes (Adams *et al*., 2016). Allegedly regressive interventions, like food taxes, are also sometimes perceived as disrespectful. Anaf *et al*. (Anaf *et al*., 2021) found that the beverage industry framed a SSB tax in Australia as a double burden to socio-economically disadvantaged people, arguing that the tax would be both ineffective and regressive. This is a position also adopted by some critical scholars and activists (Tirosh, 2014). Falbe (*Falbe*, 2020) rebuts this argument by proposing that the greater health benefit accrued to low-income people actually makes the SSB taxes progressive, rather than regressive [32].

Interventions are also accused of imposing burdens unfairly on healthy people and industry. Anaf *et al*. (Anaf *et al*., 2021) report that the soft drinks industry claims a SSB tax in Australia would unfairly impact low-income groups, non-obese people and SSB producers in an anti-Australian way [33]; it is not only unfair to scapegoat industry and its employees for the bad choices of others, but also goes against values allegedly associated with national identity. This framing reinforces a personal responsibility narrative that promotes self-care; healthy people have taken care of their own health and therefore should not have to assume the burden of the ill health of others. It also suggests that fairness, like liberty, is of special cultural relevance and serves as a powerful rhetorical tool that structures public health policy debates.

### The state’s actions must be legitimate

This theme concerns how the possession and use of power by the state is discursively legitimated. This may relate to government institutions acting within their proper mandate and legislative authority. In liberal democratic societies, the authority of the state is conceived as deriving from the consent of citizens as enacted through elections and codified in constitutional law (Beetham, 2013). This is particularly true in the US context. Falbe (Falbe, 2020) asserts that public health policies that are the result of voter choice, such as sugar taxes in California, should be considered legitimate. When a policy has not been subjected to direct democratic processes, public acceptability can be used as a proxy metric of legitimacy. Resnik (Resnik, 2015) argues that the New York soda cap would not have passed a popular vote, suggesting that it lacked legitimacy derived from probable public support [34]. This logic parallels that of representative democracy: members of the public elect officials to represent their interests and act as their proxies, who seek to understand and then enact the will of their constituents. Whether modern democracies are actually representative is debated (McCartney *et al*., 2021), however, and the relationship between policy, government, and the public is complex and multidirectional.

Government must also abide by established norms of conduct to be seen as acting legitimately. This includes abiding by its own rules, applying policy recommendations to its institutions (Kass *et al*., 2014), and acting ‘rationally’ by having reasonable goals and using accepted means to achieve them (Brooks, 2015). Institutions must act within their proper legal and political mandate as established by previous legislative or judicial precedent (Brooks, 2015; Pratt, 2015; Studdert *et al*., 2015; Coggon and Adams, 2021). Resnik (Resnik, 2015) argues that government should act ‘reasonably’ and make policies and procedures that treat like things alike and apply regulations specifically [35]. In the case of food policy more generally, this objection is used to oppose proposed action as being inconsistent across policy domains. One argument opposing SSB taxes, for example, could hold that it is illogical to treat beverages differently from other sugar-containing foods, like confectionery. Therefore, government should not intervene in taxing SSBs.

While criticism of government is not always cynical and may help craft more effective and equitable policies, practical constraints (e.g. lobbying, specific political interests of policymakers) make accusations that the government lacks coherence when it applies legislation selectively to, for example, a beverage category but not a similar food category, amount to ‘what-about-ery’ that will never realistically be satisfied. The ultimate effect of this logic would be to paralyze government action altogether due to practical and political constraints, not to mention the difficulty of agreeing on categorization.

## DISCUSSION

Our study aimed to identify from the published literature key conceptualisations of the role of the state in making DPH policies aimed at influencing individuals’ dietary practices. A total of 53 texts met our inclusion criteria, from which we identified 30 ‘thick’ accounts (Ponterotto, 2015) and randomly sampled 5 ‘thin’ texts until satisfactory information power (Malterud *et al*., 2016) was reached. Using critical reflexive thematic analysis (CRTA), we identified six themes and two subthemes that explore how the role of the state in making DPH policy is justified and challenged.

Personal responsibility frames predominated, although we also observed significant pushback from authors advocating a more socially contextualized understanding of ‘choice’. This reflects the entrenched framing of ‘lifestyle choices’ in government policy in the UK, for example, (Theis and White, 2021). Liberty was the most important value in arguing whether state intervention is justified. The utilitarian roots of public health were also apparent, with government responsible for balancing the benefits and burdens to society, members of the public, and commercial interests. The power and interests of industry were also alluded to in discussion of how to best balance the freedoms of commercial entities with the public interest in health.

Evidence-related claims, such as that an intervention does not yet have enough evidence, or that empirical evidence is so overwhelming as to compel intervention, were used to further the interests of actors attempting to influence the actions of governments. In addition to considerations about impacts to individual liberty and the distribution of benefits, the state’s legitimacy was also conceptualized as related to democratic mechanisms such as voter choice, public acceptability, and coherent policymaking. Texts also argued that the state must treat its citizens fairly, both in the application of rules and by promoting equality of outcomes and opportunities for individuals.

### Strengths and limitations

This review combines two rigorous and well-established methods, systematic searching and TA, to provide an in-depth analysis of how the role of the state in making DPH policy is justified or opposed. While our search could arguably have been expanded to additional databases, its broad reach across disciplines and perspectives provides a detailed exemplar of discourse on the legitimacy of state intervention that is not necessarily represented in conversations within the public health community. Our analysis reached sufficient information power with 35 texts, suggesting that our search was adequate to address our compass question and include key topics of relevance. It is possible that by narrowing our scope to DPH policy, we missed some arguments that are made about the role of the state in other areas or when discussing public health interventions more broadly that may also be applicable to DPH. The relevance of our findings to wider public health literature and discourse should be explored in future research.

The use of CRTA facilitated an interpretive approach that provides deep insight into the conceptual diversity of our corpus. Repeated discussion of coding and theme design within the whole author team and continuous consideration of reflexivity lend our analysis qualitative rigor. A limitation of the literature included a lack of geographic diversity of our final corpus. Only three papers (Simões, 2013; Secilmis, 2014; Tirosh, 2014) were written by authors working outside North America, Europe, Australia, and New Zealand; only one of these (Secilmis, 2014) was published outside these geopolitical confines (Turkey). Our themes reflect this context: liberty and legitimacy were defined in terms of liberal democratic processes and institutions. Our findings may therefore not be generalizable to other geopolitical contexts. We were also not able to discern whether included texts were funded or otherwise associated with industry: eighteen texts did not disclose funding or conflicts of interest (COI; Supplementary File 1), and research has shown that authors may fail to disclose COI (Wiersma *et al*., 2018; Akl *et al*., 2022). However, we believe the omission of funding statements has more to do with journalistic format (e.g. law review, analytic philosophy) and publication date than deliberate obfuscation.

### Interpretation and conclusions

Placing emphasis on liberty and personal responsibility by framing poor health as the result of ‘lifestyle choices’ maintains the spotlight on individuals and away from other actors, like government and businesses. It also parallels a history of Christian arguments about personal salvation and improvement in Western societies (Weber, 1976). Empirical and normative work in public health and philosophy has shown how these frames do not affect positive health outcomes. Promoting causal attributions of overweight and obesity to individuals is both inequitable (Friesen, 2018) and increases harmful internalized weight stigma (Pearl and Lebowitz, 2014). Personal responsibility frames also promote a flawed conception of autonomy that neglects the many barriers to full personal control over one’s circumstances and ability to ‘choose’ freely (Brown, 2013). The claim that liberty is constrained by policies that seek to modify ‘choice’ depends in part on an understanding of practices as rational, intentional, and coherent. Given evidence that dietary practices and weight are at least in some part physiologically (Martinez, 2000), genetically (Loos and Yeo, 2022), and environmentally (Schwartz *et al*., 2017) determined, consideration of whether dietary practices are instances of truly free ‘choice’ is merited. This is not to say that individuals have no control over their choices and embrace a deterministic model of human action. Rather, dietary practices should be considered as a combination of individual, institutional, and biological/physiological factors (Marteau *et al*., 2021). A conception of human agency that neglects social and biological factors is thus inadequate to characterize the autonomous (or not) character of dietary practices.

While respect for autonomy is by no means unimportant, focus on relatively insignificant decisions, like whether to purchase a soda, is less important than highly significant ‘choices’, like access to quality healthcare (Barnhill *et al*., 2014). The claim that taxation of food and drink, for example, uniquely represents a ‘slippery slope’ of increased government interference into personal life is myopic and ignores the inherent paternalism of long-game policy agendas, like tobacco control, which we find to be an acceptable role for the state (Coggon, 2020). Pricing of goods is also not a realm solely intervened upon by government. Corporations make constant pricing decisions; if setting higher prices inherently reduces individual freedom, then commercial actors are at least as responsible for reducing freedom of choice as state actors (Dobson and Hawkins, 2016). Coggon and Viens (Coggon and Viens, 2020) point out that by placing negative liberty at the heart of decision-making, as in the Nuffield ladder, we ‘double-count’ it: we use non-intervention as both the default state of policy and as a policy option. Increasingly, authors in public health and ethics oppose freedom as the most important single value to consider when discussing public health, arguing instead that values like solidarity (Jennings, 2006; Krishnamurthy, 2013) should be given more weight in moral deliberation.

Taylor (Taylor, 1971) argues that ‘common meanings’ serve as a basis for identity in human society, giving people common reference points for things that cannot necessarily be empirically demonstrated. Freedom is articulated differently by different groups, yet retains a common intersubjective relevance that everyone in a community shares (Taylor, 1971, pp. 31–32). Fairness has a similar resonance in liberal democracies, reflecting commitments to equal treatment enshrined in constitutions and underpinning welfare states. Attention to the conditions of ‘the worst off’ in society is a matter of great debate, with how a policy might affect disadvantaged people considered a key indicator of its validity. We observed similarly vague language in our review around freedom and fairness in our corpus. The predominance of fairness in public health discourse, even when it is articulated differently to serve distinct goals, is a sign that it too holds common meaning. Both freedom and fairness are ambiguous enough to appeal to different social and political groups. This ambiguity allows actors with different agendas to tap into conserved social desires and aspirations in framing their own interests (Eisenberg, 1984).

Classical liberal theory conceives of power as concentrated only in the state and Church; modern societies are much more complex. While it may fall short of the state’s ability to coerce through taxation, prohibition, and violence, corporate political power extends to other forms of ‘influence’ because of their vast resources and the reliance individuals have on the products they sell, especially in areas such as food, and their structuring of our environment. Corporations are quasi-regulatory bodies in terms of the scope of their societal influence and claim characteristics traditionally reserved for individuals, like freedom. Frameworks for analysing the influence of corporations on public health would benefit from explicitly incorporating theories of power that more accurately reflect their far-reaching influence over all aspects of society (McCartney *et al*., 2021).

This review found that language and logic shared with economics permeated all aspects of discussion about public health policy, especially through efforts to ‘balance’ health and financial savings with costs to political and commercial liberty and the encouragement of public–private partnerships. The effect of the incorporation of these logics into public health policy is to reinscribe discourses of individualism and profit, as justified by an ethic of utility maximization. A dominant discourse of evidence-based policy raises the evidential bar for action ever higher, becoming an evidence-deterministic mode of thinking that neglects the precautionary principle in favour of a solely risk-based approach (Martuzzi, 2007).

In a recent article, Maani *et al*. (Maani *et al*., 2022) called for the public health community to do a better job of framing the social determinants of health to combat personal responsibility narratives that lead to less effective policy interventions. They identified the ‘pollution’ of public health discourse by pro-industry actors and its implicit embeddedness in our public consciousness as a threat to public health: ‘All too often, the choices we make about what we say and how we say it are influenced by and reflect corporate interests’ (Maani *et al*., 2022, p. 1). They advocate for greater transparency and accountability for COIs as well as caution when encountering industry frames. Our analysis additionally identifies frames that benefit industry and prevent effective regulation entrenched in our academic literatures. We must be more aware of how importing language from economics comes with strings attached: when we normalize cost-benefit analysis, we also normalize the superiority of associated logics of financial benefit, markets, and rational consumers. More care is necessary to ensure that public health avoids ‘pollution’ by implicitly pro-industry framings through unreflexively embracing the languages and logics of free market economics.

## Supplementary Material

daad100_suppl_Supplementary_Material
