# Supplementary material for: Understanding the role of the state in dietary public health policymaking: a critical scoping review
Source: Health Promot Int. 2023 Sep 4;38(5):daad100. doi: 10.1093/heapro/daad100 (PMC10476878; doi:10.1093/heapro/daad100)
Supplement: daad100_suppl_Supplementary_Material [file daad100_suppl_supplementary_material.zip › Supplemental File 2 - Search strategy.docx]

## Supplemental File 2: Search strategy

All databases were searched in January 2021. The following journals were also hand-searched in February and March 2021:

- *Public Health Ethics* (April 2008 – November 2020)
- *Journal of Medical Ethics* (March 1975 – March 2021)
- *Journal of Public Health* (February 1979 – December 2020)
- *Health Care Analysis* (June 1993 – March 2021)

| DATABASE | SEARCH STRING | HITS | NOTES |
| --- | --- | --- | --- |
| Scopus | (TITLE-ABS-KEY ( ( nanny  OR  ( role  W/3  ( government  OR  state ) )  OR  paternalis*  OR  ( freedom*  OR  libert* )  AND  ( "public health"  AND  ( polic*  OR  regulat*  OR  program* ) ) ) ) )  AND  ( LIMIT-TO ( LANGUAGE ,  "English" ) ) | 2,518 |  |
| MedLine (OVID) | 1: (nanny or role) adj3 (state or government)  2: paternalis*  3: freedom* or libert*  4: "public health"  5: polic* or regulat* or program*  6: 1 or 2 or 3  7: 4 and 5  8: 6 and 7  9: limit 8 to English language | 1,288 |  |
| PhilPapers | (nanny \| (role NEAR:3 (state \| government)) \| paternalism \| libertarian \| freedom \| choice) & ("public health" \| "population health") | 160 | 4 non-English titles manually removed. |
| Web of Science | ((AB=((nanny OR (role NEAR/3 government OR role NEAR/3 state) OR paternalis* OR (libert* or freedom*) ) AND (public health AND (polic* OR regulat* OR program*) ))))  AND LANGUAGE: (English)    Timespan: All years. Indexes: SCI-EXPANDED, SSCI, A&HCI, CPCI-S, CPCI-SSH, BKCI-S, BKCI-SSH, ESCI, CCR-EXPANDED, IC. | 1,069 |  |
| PsycINFO | AB ((nanny OR (role N3 government OR role N3 state) OR paternalis* OR libert* OR freedom*) AND ("public health" AND (polic* or regulat* or program*))) | 266 |  |
| ProQuest | AB((nanny OR (role NEAR/3 government OR role NEAR/3 state) OR paternalis* OR freedom* OR libert*) AND ("public health" AND (polic* or regulat* or program*)))    NOT news, fiction, obituary    English only | 1,296 | Excluding magazines, artistic/aesthetic works, trade journals not relevant to public health, and newspapers |
| OSF Preprints | nanny OR "role government"~3 OR "role state"~3 OR paternali* OR libert* OR freedom* AND "public health" AND (program* OR regulat* OR polic*) | 14 | Excluded non-English titles; did not add duplicates |
| WorldCat | Key words: nanny state public health  Language: English  Type: Books, e-books, dissertations  Library: any  Date: any | 41 | 2 duplicates removed |
| SSRN | paternalism “public health”  libertarian “public health”  nanny state “public health” | 24  5  0 | Very limited search ability; did not add duplicates |
| PhilSci Archive | Abstract, any of: nanny state government paternalism libertarianism  Keywords, all of: health | 5 | Very limited search ability; did not add duplicates |
| Google Scholar | “nanny state”\|”role of the state”\|”role of the government”\|libertarian\|paternalism\|freedom “public health” policy\|regulation\|program | 36 | First 100 results scanned and 36 included; did not add duplicates |
| Google | “nanny state”\|”role of the state”\|”role of the government”\|libertarian\|paternalism\|freedom “public health” policy\|regulation\|program | 26 | First 100 results scanned and 26 included; did not add duplicates |
| *Public Health Ethics* | Hand searching. April 2008 – November 2020. | 25 |  |
| *Journal of Medical Ethics* | Hand searching. March 1975 – March 2021. | 80 |  |
| *Journal of Public Health* | Hand searching. February 1979 – December 2020. | 14 |  |
| *Health Care Analysis* | Hand searching. June 1993 – March 2021. | 54 |  |
