# Supplementary material for: Understanding the role of the state in dietary public health policymaking: a critical scoping review
Source: Health Promot Int. 2023 Sep 4;38(5):daad100. doi: 10.1093/heapro/daad100 (PMC10476878; doi:10.1093/heapro/daad100)
Supplement: daad100_suppl_Supplementary_Material [file daad100_suppl_supplementary_material.zip › Supplemental File 3 - PRISMA flow diagram.docx]

# Supplemental File 3 – PRISMA flow diagram

Texts classed as ‘thin’:

Not focussed on dietary public health (n = 23)

Records identified from:

Databases (n = 6,686)

Websites (n = 62)

Hand searching (n = 173)

Records removed before screening:

Duplicate records removed (n = 2,695)

**Identification**

Records screened

(n = 4226)

Records excluded

(n = 3572)

Texts retrieved

(n = 632)

Texts assessed for eligibility

(n = 632)

**Screening**

Texts excluded:

Not justifying, challenging, or explaining the role of the state (n = 272)

Not health-affecting indiv. practices (n = 52)

High-risk groups only (n = 29)

Inappropriate format (n = 13)

Duplicate (n = 8)

Not in English (n = 4)

Texts included in review

(n = 254)

Texts excluded:

Not focussed on dietary practices (n = 201)

**Included**

Texts included on dietary practices (n = 53)

**Corpus**

**Narrowing focus**

**Ing focus**

Texts classed as ‘thick’

(n = 30)

Final corpus

(n = 35)

Texts classed as ‘thin’ and randomly sampled until information power (n = 5)

From: Page MJ, McKenzie JE, Bossuyt PM, Boutron I, Hoffmann TC, Mulrow CD, et al. The PRISMA 2020 statement: an updated guideline for reporting systematic reviews. BMJ 2021;372:n71. doi: 10.1136/bmj.n71
