# Supplementary material for: Understanding the role of the state in dietary public health policymaking: a critical scoping review
Source: Health Promot Int. 2023 Sep 4;38(5):daad100. doi: 10.1093/heapro/daad100 (PMC10476878; doi:10.1093/heapro/daad100)
Supplement: daad100_suppl_Supplementary_Material [file daad100_suppl_supplementary_material.zip › Supplemental File 4 - Reflexivity statement and note on terminology.docx]

## Supplemental File 4: Reflexivity statement and note on terminology

### Reflexivity

Reflexivity refers to both the recognition of how the subjectivity of researchers shapes our work and how we reflect on this as a part of our research process. Our understanding of concepts like choice and liberty is shaped by our situated personal and academic experience. As researchers in public health, we bring our own conceptions of what makes for “good” policy into our research. The same can be said for particular justifications for public health involving health benefit, equity, and personal agency. JA and MW have previously published research on the equity and efficacy of population vs high risk public health interventions (Adams et al., 2016; Coggon and Adams, 2021) and co-lead a population health research group that focuses on lower agency public health interventions. Our analysis necessarily reflects these personal and professional investments. Our reflexivity practice included a journal kept by lead author (NK) throughout the research process and co-writing of this section.

### A note on terminology

Choice, liberty, freedom, and autonomy are central to debates about the legitimate role of the state. These terms are, however, not epistemologically interchangeable and carry distinct philosophical commitments and historical connotations. *Autonomy* refers to the capacity for self-governance, of which philosophers debate the necessary and sufficient conditions (Buss and Westlund, 2018). *Liberty* is the condition of being free, whereas autonomy is the capacity to act freely. Isaiah Berlin’s conception of “negative liberty” (“freedom from”) is at the heart of many arguments in public health, including the “nanny state” debate. In this formulation, liberty is understood as freedom from external constraints (Berlin, 1969). Berlin’s conception does not rely on preference, achievability, or the desirability of options: it is strictly concerned with the possibility of choice. Free-market economic theory also holds choice and respect for the liberty of individuals and commercial actors as central tenets. The terminology used to detail reductions, increases and modifications of choice in our corpus is variable: authors used *freedom*, *liberty*, *choice*, and *autonomy* at times interchangeably to refer to the decisions and capacity for decision-making of individuals. We use *liberty* and *freedom* interchangeably to refer to the condition of being free, reserving *autonomy*, personal *agency*, and *choice* to describe the ability to make decisions.

### References

Adams, J., Mytton, O., White, M., Monsivais, P., 2016. Why Are Some Population Interventions for Diet and Obesity More Equitable and Effective Than Others? The Role of Individual Agency. PLoS Med. 13, e1001990–e1001990. https://doi.org/10.1371/journal.pmed.1001990

Berlin, I., 1969. Two concepts of liberty. Oxford University Press, Oxford.

Buss, S., Westlund, A., 2018. Personal Autonomy, in: Zalta, E.N. (Ed.), The Stanford Encyclopedia of Philosophy. Metaphysics Research Lab, Stanford University.

Coggon, J., Adams, J., 2021. ‘Let them choose not to eat cake...’: Public health ethics, effectiveness and equity in government obesity strategy. Future Heal. J 8, 49–52. https://doi.org/10.7861/fhj.2020-0246
