# Supplementary material for: Understanding the role of the state in dietary public health policymaking: a critical scoping review
Source: Health Promot Int. 2023 Sep 4;38(5):daad100. doi: 10.1093/heapro/daad100 (PMC10476878; doi:10.1093/heapro/daad100)
Supplement: daad100_suppl_Supplementary_Material [file daad100_suppl_supplementary_material.zip › Supplemental File 5 - Included texts.docx]

## Supplemental File 5 – Included texts

| Title | Author(s) | Author location(s) | Date | Discipline | Topic | Designated as thick or thin | Brief summary | Funding disclosed? |
| --- | --- | --- | --- | --- | --- | --- | --- | --- |
| Sweet talk': framing the merits of a sugar tax in Australia | Anaf et al. | Australia | 2021 | Public health | SSB taxes | Thick | Empirical analysis of framing of a SSB tax in Australia with four themes: economic impacts, equality, obesity, and "nanny state" versus individual liberty. | Yes |
| The Value of Unhealthy Eating and the Ethics of Healthy Eating Policies | Barnhill et al. | USA | 2014 | Public health ethics | General public health | Thick | Normative discussion of justifiability of policies targeting food and eating depends on the (dis)value of particular "eating experiences." | Yes |
| Personal responsibility or shared responsibility: What is the appropriate role of the law in obesity prevention? | Brooks | Australia | 2015 | Law | General public health | Thick | “Invasive” public health intervention justified by public interest case and to correct market failure. | No |
| Promoting Justice and Autonomy in Public Policies to Reduce the Health Consequences of Obesity | Buchanan | USA | 2015 | Public health ethics | General public health | Thick | Broad discussion of autonomy in public health policy. | No |
| ‘Let them choose not to eat cake...’: Public health ethics, effectiveness and equity in government obesity strategy | Coggon & Adams | UK | 2021 | Public health ethics | General public health | Thin | Provides conditions for lower agency, equitable public health interventions. | Yes |
| Cheeseburgers, Race, and Paternalism: Los Angeles’ Ban on Fast Food Restaurants | Creighton | USA | 2009 | Law | Zoning regulations | Thick | Opposes Los Angeles fast food retail ban as ineffective, unfair, and unjustly paternalistic | No |
| The ethics of excise taxes on sugar sweetened beverages | Falbe | USA | 2020 | Public health ethics | SSB taxes | Thick | Evaluates SSB taxes and finds them justified on various grounds; industry should share responsibility for health outcomes. | Yes |
| Ethical Considerations for Food and Beverage Warnings | Grummon et al. | USA | 2020 | Public health | Food warning labels | Thick | Food warning labels are ethically justifiable across various considerations. | Yes |
| Obesity, liberty, and public health emergencies | Herington, Dawson & Draper | USA, Australia, UK | 2014 | Philosophy | General public health | Thick | Obesity has some of the morally relevant features of a public health emergency, which provides justification for intervention. | No |
| What’s Wrong with Mandatory Nutrient Limits? Rethinking Dietary Freedom, Free Markets and Food Reformulation | Kaldor | Australia | 2018 | Public health ethics | Nutrient limits | Thin | Public health ethics is poorly equipped to deal with differences between regulatory modes; in this case, mandatory versus voluntary nutrient reformulation. Argues should be treated more similarly. | Yes |
| Ethics and Obesity Prevention: Ethical Considerations in 3 Approaches to Reducing Consumption of Sugar-Sweetened Beverages | Kass et al. | USA | 2014 | Public health ethics | SSB policies | Thick | Assesses ethical validity of three American SSB policies via six “considerations” and challenges the idea that SSB choice is an example of “free” choice. | Yes |
| The harm of bioethics: A critique of Singer and Callahan on obesity | Mayes | Australia | 2015 | Philosophy | General public health | Thick | Strategies targeting individuals - in this case, stigmatisation and increased cost to individual based on weight - are not justified; they actually contribute to harm. | No |
| Legal and policy approaches to the obesity epidemic | Mello | USA | 2012 | Medicine | General public health | Thick | Government intervention into obesity is justified by obesity’s socially-determined nature, paternalism and solidaristic obligations. | No |
| Obesity - Personal choice or public health issue? | Mello | USA | 2008 | Public health | General public health | Thick | Intervention in obesity is justified by impaired autonomy; moral reasons, including solidarity; and social costs. | Yes |
| Evaluating the Legitimacy of Contemporary Legal Strategies for Obesity | Morain | USA | 2015 | Philosophy | General public health | Thick | Rawls' principle of legitimacy is a better tool for justifying public health interventions than the harm principle. | Yes |
| Child Rights as a Basis for the Regulation of Food Marketing: The Role of the un Convention on the Rights of the Child | Ó Cathaoir | Denmark | 2017 | Public health | Advertising restrictions | Thick | The UN Convention on the Rights of the Child (UNCRC) can provide a rights-based justification for HFSS food advertising restrictions. | No |
| Is the obesity epidemic a public health problem? A review of Zoltan J. Acs and Alan Lyles’s obesity, business and public policy | Philipson & Posner | USA | 2008 | Economics | General public health | Thick | Overviews justification for various policies to address obesity from an economics perspective. | No |
| Limiting Liberty to Prevent Obesity: Justifiability of Strong Hard Paternalism in Public Health Regulation Response | Pope | USA | 2014 | Law | General public health | Thick | Hard paternalism can be justified as a way to address obesity. | No |
| The Limits of Anti-Obesity Public Health Paternalism: Another View | Pratt | USA | 2014 | Law | New York soda cap | Thin | Overviews Friedman's (2014) account of the defeat of the New York soda cap policy and proposes an alternative view of how policy failed: the “new” public health paternalism is difficult for policy actors to justify. | No |
| Paternalism, autonomy, and food regulation | Priest | USA | 2015 | Public policy | New York soda cap | Thick | Explores two different justifications for the New York soda cap: the harm principle and paternalism. | No |
| Raising healthy children: Moral and political responsibility for childhood obesity | Purcell | Canada | 2010 | Public health | Children's health | Thick | UNCRC implies an obligation of state to provide for children's health; individualistic public health strategies do not meet this challenge. | No |
| Food and Beverage Policies and Public Health Ethics | Resnik | USA | 2015 | Philosophy | General public health | Thick | Describes how an intervention into food/beverage consumption can be ethical: policy must serve important social goals, be likely to be effective, less burdensome options are not likely to be as effective, and be fair. | Yes |
| Trans Fat Bans and Human Freedom | Resnik | USA | 2010 | Philosophy | Trans fat bans | Thick | Banning trans fats is an unacceptable intrusion on liberty and signals a slippery slope to further unwarranted state intervention. | Yes |
| Paternalistic Food and Beverage Policies: A Response to Conly | Resnik | USA | 2014 | Public health ethics | General public health | Thick | Paternalistic health interventions on food and beverages can be justified under certain key conditions: they must address significant health problem, evidence of their efficacy must exist, and less restrictive alternatives are likely to be ineffective. | Yes |
| Taxation and Economic Incentives on Health-Related Commodities: Alcohol, Tobacco and Food | Sassi et al. | OECD | 2014 | Public health | Taxes | Thick | Provides rationale for taxation of tobacco, alcohol, and food. | No |
| The economics of obesity and the role of government in obesity prevention: The Turkish case | Secilmis | Turkey | 2014 | Economics | General public health | Thick | Considers role of government to intervene in obesity on economic grounds. | No |
| Evidence v. rights-based decision making for nutrition | Shrimpton | UK | 2003 | Public health | General public health | Thick | The role of the state is to respect, promote, and facilitate human rights, including the right to adequate nutrition. | No |
| Paternalism and health law: Legal promotion of a healthy lifestyle | Simões | Macau | 2013 | Law | General public health | Thick | Overviews ethical issues at stake in health law, and whether food policies can be justified (and are or are not paternalistic). | No |
| Searching for Public Health Law's Sweet Spot: The Regulation of Sugar-Sweetened Beverages | Studdert, Flanders & Mello | USA | 2015 | Public policy | SSB taxes | Thin | Overviews arguments opposing SSB taxation and provides counterarguments. | Yes |
| Ethics and prevention of overweight and obesity: an inventory | ten Have et al. | The Netherlands, Portugal | 2011 | Public health | General public health | Thick | Ethical framework for obesity policies/programs. | Yes |
| An ethical framework for the prevention of overweight and obesity: a tool for thinking through a programme's ethical aspects | ten Have et al. | The Netherlands | 2012 | Public health | General public health | Thick | Ethical framework for obesity policy/programs. Provides eight questions to guide design. | Yes |
| Three Comments on Paternalism in Public Health Response | Tirosh | Israel | 2014 | Law | General public health | Thin | Debates wrongs of paternalism in conversation with Friedman (2014) article. | No |
| Sugar, Taxes, & Choice | Veliz et al. | UK, USA | 2019 | Public policy | SSB taxes | Thick | SSB taxes are permissible and do not undermine autonomy. | Yes |
| Obesity, equity and choice | Wilkinson | New Zealand | 2019 | Philosophy | General public health | Thick | Interventions to promote equity that are justified by correcting bad options or bad choices for the worst-off are not yet justifiable based on evidence. | Yes |
| Obesity and Health System Reform: Private vs. Public Responsibility | Yang & Nichols | USA | 2011 | Public health | General public health | Thick | There is a social interest in curbing obesity costs because costs are not borne by obese individuals alone; evidence-based interventions that can reduce these costs may be justified. | No |
