# Supplementary material for: Understanding the role of the state in dietary public health policymaking: a critical scoping review
Source: Health Promot Int. 2023 Sep 4;38(5):daad100. doi: 10.1093/heapro/daad100 (PMC10476878; doi:10.1093/heapro/daad100)
Supplement: daad100_suppl_Supplementary_Material [file daad100_suppl_supplementary_material.zip › Supplemental File 6 - Summary of analytic themes.docx]

## Supplemental File 6 – Summary table of analytic themes

| Theme name | Description |
| --- | --- |
| The state should not intervene in choice | Whether it is acceptable to limit individuals’ liberty (or autonomy, freedom, or choice) to achieve public health benefits, and if so, to what degree and through what mechanisms. Includes nanny state and other objections about state paternalism. |
| Responsibility for health | Encompasses arguments about what and who are responsible for poor health outcomes. Frames both problem and solution: whether the state is justified in intervening depends on what causal model is reported as responsible and to whom responsibility is attributed. |
| The state should protect the public | State cast as a protector of certain key valuable things: health, rights, and the public interest. Also concerns what constitutes a ‘public’ problem. |
| The state should partner with industry | Industry either must or can be involved in partnership with the state to improve health. Debate over public-private partnerships and voluntary (co-)regulation is also included. |
| The state must balance benefits and burdens | Weighing-up or balancing of burdens with benefits of interventions is the responsibility of the state. Sometimes referred to as proportionality. Often using language from economics/finance of ‘costs’ and ‘externalities’. |
| The state must provide evidence of efficacy | Using previous research or outcomes to justify/oppose further interventions - both health outcomes and cost-benefit financial outcomes. An evidence threshold must be met; evidence must be ‘substantial’ enough. Also used to frame the problem – e.g., descriptive statistics about obesity and diabetes rates. |
| The state must act fairly | Must treat parties subject to regulation equally and/or equitably. Related to social justice and to procedural norms of a legitimate state. |
| The state’s actions must be legitimate | Discusses where the state draws its authority from, as well as how actions are considered legitimate or illegitimate. |
