# Supplementary material for: Understanding the role of the state in dietary public health policymaking: a critical scoping review
Source: Health Promot Int. 2023 Sep 4;38(5):daad100. doi: 10.1093/heapro/daad100 (PMC10476878; doi:10.1093/heapro/daad100)
Supplement: daad100_suppl_Supplementary_Material [file daad100_suppl_supplementary_material.zip › Supplemental File 7 - Selected quotations used for analysis.docx]

# Supplemental File 7: Selected quotations from included texts used for analysis

1. The prevailing political ideology rejects paternalistic intervention by the state and emphasizes individual autonomy, which provides freedom to make ‘wrong’ choices, so long as they do not harm others. The economic expression of this philosophy is market individualism—defined as confidence in the free market as a regulatory mechanism and belief in the importance of allowing individuals to make choices within the market and accept responsibility for consequences that arise from them. (Mello, 2008, p. 2)
2. On the contrary, adopting a negative approach, by increasing taxes on specific unhealthy foods or ingredients, can be considered paternalistic as it curtails the gastronomical liberty of citizens with the goal of forcing upon them a healthy lifestyle without their consent. A paternalistic tax policy would be very hard to explain, as Tax Law is built on principles such as legitimacy, proportionality and fairness. A paternalistic tax would probably be the worst form of paternalism. (Simões, 2013, p. 363).
3. Our analysis identifies both ethical strengths and weaknesses of food warnings, including that: 1) warnings are likely to generate important benefits including increased consumer understanding and informed choice, healthier purchases, and potential reductions in obesity prevalence; 2) warnings evoke negative emotional reactions, but these reactions are an important mechanism through which food warnings encourage healthier behaviors and promote informed choice; 3) warnings appear unlikely to have ethically unacceptable effects on social and cultural values, attributions of responsibility, liberty, or privacy. (Grummon et al., 2020, p. 1)
4. Examples of obesity strategies that require less individual agency include taxes on sugary drinks, restrictions on advertising of less healthy foods, and planning controls on where new takeaway outlets can open. These are all hypothesised to achieve their effects not through deliberative mechanisms whereby consumers weigh up information on risks and benefits and make informed ‘choices’, but by changing the fiscal, social and physical environment in which individuals live so that the healthier options become not just easier, but the default. (Coggon and Adams, 2021, p. 50)
5. Because decisions about food are often made without active thinking, helping consumers keep products’ health consequences at top of mind when making a purchase decision is likely to enhance their capacity to make and carry out informed choices. (Grummon et al., 2020, p. 6)
6. Where an agent’s behavior poses a grave risk to his or her own well-being, we have a reason to suspect that the capacity for autonomous choice is compromised (perhaps by a lack of information or a momentary impairment). (Herington et al., 2014, p. 28)
7. The first challenge we raise is whether our consumption behavior, absent policy intervention, is truly free and unconstrained. Research studies have revealed that our ordinary eating behavior, absent any public policy intervention, may be more constrained than we sometimes imagine. Data increasingly highlight how sensitive our individual eating behavior is to innumerable, even small, external influences in our environment. (Kass et al., 2014, p. 792)
8. Although these policies may have a positive impact on human health, they open the door to excessive government control over food, which could restrict dietary choices, interfere with cultural, ethnic, and religious traditions, and exacerbate socioeconomic inequalities. These slippery slope concerns cannot be dismissed as far-fetched, because the social and political pressures are in place to induce additional food regulations. To protect human freedom and other values, policies that significantly restrict food choices, such as bans on types of food, should be adopted only when they are supported by substantial scientific evidence and when policies that impose fewer restrictions on freedom, such educational campaigns and product labeling, are likely to be ineffective. (Resnik, 2010, p. 8)
9. Selling a product is not an inherently freer enterprise than promoting health through public policy, and it seems unfair to demand more restrictions from the state than from businesses when the state’s objectives reflect a concern for the well-being of its citizens. (Veliz et al., 2019, p. 29)
10. A final point regarding liberty is one of fairness over who—industry or government—may influence consumer choice, particularly when industry actions reflect a profit motive and government actions “reflect a concerns for the well-being of its citiznes.” [23] As Schwartz et al. [74] note, “...retailers and manufacturers are already influencing consumers in ways that are detrimental to their health, and if it is ethical to use strategies to sell more unhealthy products, it is certainly ethical to use alternative strategies to sell fewer.” (Falbe, 2020, p. 4)
11. Exposure to food advertising, the inaccessibility of healthy eating options, low socioeconomic status, and poor educational attainment are all strong influences on an individual’s risk of becoming obese. In this sense, individuals are exposed to many behaviors and structures that increase the risk that they will become obese. It is simply untrue that most individuals who are obese would choose to remain obese and that they freely engage in behaviors that they know will make them obese. We ought to recognize that structures, behaviors, and individual acts that raise the risk that an individual will act contrary to his or her professed interests may also be legitimate targets of intervention. (Herington, 2014, p. 33)
12. While the marketing and advertising conducted by food and beverage companies may encourage people to make unhealthy dietary decisions, individuals should still be held accountable for their own free choices. (Resnik, 2015, pp. 130–131)
13. The participant expanded on this argument, stating that attributing obesity to environmental factors undermines measures to augment individual responsibility: There are so many contributing factors to obesity. One of them is clearly, for want of a better word, indolence...Attributing lifestyle disease to social, cultural, legislative and environmental factors erodes doctors’ ability to improve patients’ health literacy and selfcare. (Think Tank participant #1) (Anaf et al., 2021, p. 1340)
14. While public health advocates can play an essential role in counteracting the influence of industry, which tends to undermine public health in the pursuit of profits, the most reasonable way forward is to develop policies that provide a fair balance between public health and other values. (Resnik, 2014, p. 174)
15. Two grounds for regulation are particularly persuasive, and cut through the discourse of personal responsibility. The first and most immediate justification is that governments owe a responsibility to their respective polities to monitor and manage the collective health of the population. This is an ethical imperative, and an expectation that citizens hold of their governments. (Brooks, 2015, p. 113)
16. Even if a fat tax drew high public approval and effectively promoted health, it would be morally and legally unjustifiable. In legal terms, such a policy should be declared unconstitutional because it would infringe on individual dignity, privacy, and autonomy. (Tirosh, 2014, p. 1803)
17. The basis for regulation here is that advertising has a particularly pronounced effect on the consumption patterns of vulnerable audiences, who are incapable of critically evaluating the advertised product on nutritional grounds. (Brooks, 2015, p. 117)
18. One could argue that school policies are ethically justified because they serve an important goal (i.e. promoting children’s health), they are effective at achieving this goal, and less burdensome means (such as providing children with nutritional information) are not likely to be very effective. Although these policies significantly restrict children’s food and beverage choices, one could argue that this limitation on autonomy is warranted because children are not fully autonomous decision-makers, and their rights to autonomy may be limited appropriately [3]. (Resnik, 2015, p. 128)
19. Parents and governments alike need to recognize that their choices affect children, as developing citizens, and that children possess rights to protection from preventable harms, even when it limits parental authority. Health is not only a private concern; it is deeply affected by public and commercial interests, which must be managed and regulated to prevent harm to children and to support families. (Purcell, 2010, pp. 440–441)
20. Solidarity is the notion that individuals should not be left by their community to bear terrible burdens alone; we have a moral duty to prevent suffering where we can. […] It could be argued, therefore, that as a representative of the community, the state must intervene to avoid these harms where possible. (Mello, 2008, p. 2)
21. If we take rights seriously, we have in the Convention a potentially powerful legal tool through which to challenge the factors leading to wide-spread childhood obesity and chronic disease. These factors clearly violate the rights of children, according to the Convention. Providing children with poor nutrition and limited physical activity in an 'obesogenic' environment fails to uphold the 'best interests' principle for children. Because obesity can have a powerful impact on a child's future (physically, emotionally, economically), adults must strive to raise healthy children, but they may need help from a changed environment.1 The legal obligations of the Convention dictate that parents, government, industry, and other citizens all have a responsibility to ensure children's rights are upheld. (Purcell, 2010, p. 441)
22. Quite the contrary, the evidence shows that the food industry has begun to eliminate the use of artificial trans fats in response to consumer preferences. The FDA requires that food labels include information about the nutritional content of foods and beverages, including trans fats content. To attract consumers, many manufacturers also prominently indicate on their packaging that their product has ‘no trans fats’ or ‘zero trans fats’. One might argue that trans fat bans are an unnecessary use of government coercion because companies have started voluntarily eliminating these products (Resnik, 2010). (Resnik, 2014, p. 175)
23. Critics argue that voluntary food industry schemes are doomed to fail because there is ‘no evidence for an alignment of public health interest in curbing obesity with that of the food and beverage industry’ (Stuckler and Nestle, 2012: 2). (Kaldor, 2018, p. 85)
24. The main reason for public intervention in this regard is the impact of the cost of overweight-related diseases on the public health system. The economic analysis indicates that there are externalities associated with overweight and obesity. This takes the problem beyond the individual and makes it a legitimate focal point of public concern. (Simões, 2013, p. 362)
25. Our analysis of available evidence suggests that food warnings may be ethically defensible, depending upon the nature, magnitude and distribution of their costs and benefits. (Grummon at al., 2020, p. 10)
26. Finally, he closes with a broader normative claim that public health regulators ‘should pursue all solutions open to them’ and rationalize their public health regulations with cost-benefit analyses. (Pratt, 2015, pp. 1909–1910)
27. Furthermore, proportionality requires programs whose burdens remain high to provide greater benefits; related, the quality and magnitude of evidence supporting a program’s purported benefit should increase when risks or burdens are greater. (Kass et al., 2014, p. 788)
28. Various effective programmes are being implemented to reduce overweight. However, the (cost-) effectiveness of programmes to prevent overweight is often unknown, unfavourable or hard to prove. (ten Have et al., 2013, p. 300)
29. Our analysis suggests that many individual and targeted strategies are ethically defensible and, in the short term, undoubtedly should be implemented. And yet, in the long run, a more widespread policy requirement to use government funds in ways consistent with evidence-based and periodically revised government guidance is not only likely to achieve a more comprehensive benefit, but also is the only way to invoke—in perception and in reality—our commitments to fairness and social justice as we work to combat obesity—and to public health more broadly—in the United States. (Kass et al., 2014, p. 793)
30. The industry position framed a [SSB] tax as an ineffective ‘silver bullet’ solution to a complex problem and promoted behavioural reform. The public health perspective instead framed it as just one of multiple strategies, including behavioural change, that are part of broader strategies targeting the food environment. (Anaf et al., 2021, p. 1338)
31. On the contrary, adopting a negative approach, by increasing taxes on specific unhealthy foods or ingredients, would raise the global price of food (assuming that healthy food prices remained the same) and thus harden the access of less affluent people to healthy products. (Simões, 2013, p. 360)
32. Tax opponents have focused on distribution of money, arguing that SSB taxes are regressive (i.e., result in a higher cost burden for low-income than high-income individuals) and are therefore unfair. Whereas, tax proponents emphasize distribution of health and social benefits, arguing that SSB taxes are progressive (i.e., result in a greater distribution of good health, educational opportunities, and other benefits to low-income individuals) thereby promoting equality (Falbe, 2020, p. 3)
33. As one respondent argued: “A sugar tax is not consistent with the Australian concept of fairness and equal treatment by our public health system. Australians do not regard sugar consumption as a bad thing or guilty pleasure, or would probably not accept the notion that they should pay extra for the enjoyment...Our universal health system does not discriminate against fat [sic] people or any other body shape. Rather, our system is purposely designed not to discriminate against anyone.” (Think Tank participant #1) (Anaf et al., 2021, p. 1339)
34. Third, the ban was procedurally unfair because it was imposed by the city government even though most residents opposed it. The ban probably would have failed if put to a vote [21]. (Resnik, 2015, p. 130)
35. Third, trans fat bans raise signiﬁcant fairness issues, because consuming artiﬁcial trans fats may be no more risky than eating other foods linked to heart disease or cancer, such as processed meats and foods high in saturated animal fat. A commitment to fairness implies treating all foods equally with regard to risks: if artiﬁcial trans fats should be banned, then perhaps hot dogs, heavy cream, and other foods should be banned as well. (Resnik, 2015, p. 129)
